# Crystal structure of *N*,*N*′-di­benzyl­pyromellitic diimide

**DOI:** 10.1107/S2056989016017710

**Published:** 2016-11-15

**Authors:** Hansu Im, Suk-Hee Moon, Tae Ho Kim, Ki-Min Park

**Affiliations:** a Research Institute of Natural Science and Department of Chemistry, Gyeongsang National University, Jinju 52828, Republic of Korea; b Department of Food and Nutrition, Kyungnam College of Information and Technology, Busan 47011, Republic of Korea

**Keywords:** crystal structure, pyromellitic di­imide derivative, hydrogen bonding, two-dimensional network

## Abstract

The title compound, C_24_H_16_N_2_O_4_ [systematic name: 2,6-di­benzyl­pyrrolo­[3,4-*f*]iso­indole-1,3,5,7(2*H*,6*H*)-tetra­one], consists of a central pyromellitic di­imide moiety with terminal benzyl groups at the N-atom positions. The mol­ecule is located about an inversion centre, so the asymmetric unit contains one half-mol­ecule. In the mol­ecule, both terminal phenyl groups, tilted by 72.97 (4)° with respect to the mean plane of the central pyromellitic di­imide moiety (r.m.s. deviation = 0.0145 Å), are oriented away from each other, forming an elongated S-shaped conformation. In the crystal, mol­ecules are connected *via* weak C—H⋯O hydrogen bonds and C—H⋯π inter­actions, resulting in the formation of supra­molecular layers extending parallel to the *ab* plane.

## Abstract 2

The title compound, C_24_H_16_N_2_O_4_ (systematic name: 2,6-di­benzyl­pyrrolo­[3,4-*f*]iso­indole-1,3,5,7(2*H*,6*H*)-tetra­one), lies about a crystallographic inversion center at the center of the pyromellitic di­imide moiety which is planar. In the crystal, inter­molecular C—H⋯O hydrogen bonds and C—H⋯π inter­actions lead to the formation of a two-dimensional supra­molecular network.

## Chemical context   

As a result of their potential applications in organic photovoltaics (Huang *et al.*, 2014 ▸) and as mol­ecular electronic devices (Guo *et al.*, 2014 ▸) and energy storage devices (Song *et al.*, 2010 ▸), several π-conjugated, redox-active aromatic di­imides including pyromellitic di­imides, naphthalene di­imides and perylene di­imides have received considerable attention from materials chemists. Additionally, π-conjugated aromatic di­imides and their derivatives are used as rigid structural components in supra­molecular assemblies for the exploitation of supra­molecular inter­actions such as hydrogen-bonding and halogen–π inter­actions (Hay & Custelcean, 2009 ▸; Lu *et al.*, 2007 ▸; Gamez *et al.*, 2007 ▸). Recently, our group reported a copper(I) coordination polymer with a pyromellitic di­imide ligand, namely *N*,*N*′-bis­[3-(methyl­thio)­prop­yl]pyromellitic di­imide, and revealed the presence of halogen–π inter­actions between the chlorine atoms of a di­chloro­methane solvent mol­ecule of crystallization and pyromellitic di­imide rings (Park *et al.*, 2011 ▸). In an extension of our studies of pyromellitic di­imide derivatives, we have prepared the title compound by the reaction of pyromellitic dianhydride with 2-phenyethyl­amine and we report its crystal structure here.

## Structural commentary   

**Figure 1:**
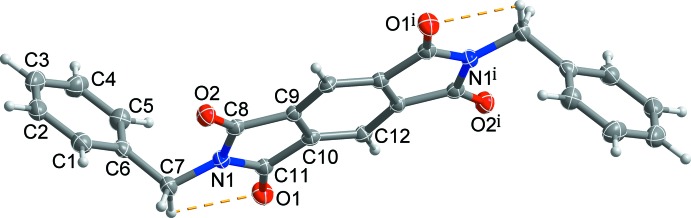
A view of the mol­ecular structure of the title compound, showing the atom-numbering scheme. Displacement ellipsoids are drawn at the 50% probability level. H atoms are presented as small spheres of arbitrary radius and yellow dashed lines represent the intra­molecular C—H⋯O short contacts. [Symmetry code; (i) −*x* + 2, −*y* + 1, −*z*.]

The mol­ecular structure of the title compound consists of a central pyromellitic di­imide ring system with terminal benzyl groups on each of the inversion-related nitro­gen atoms (Fig. 1 ▸). As the mol­ecule is located about a crystallographic inversion centre, the asymmetric unit of the compound comprises one half-mol­ecule. Short intramolecular C—H⋯O contacts (Table 1 ▸) enclose *S*(5) rings and may contribute to the planarity of the pyromellitic di­imide ring system (r.m.s. deviation = 0.0145 Å). The two terminal phenyl groups in the mol­ecule are oriented away from each other, forming an elongated S-shaped conformation. The terminal phenyl ring is tilted by 72.97 (4)° with respect to the mean plane of the central pyromellitic di­imide moiety.
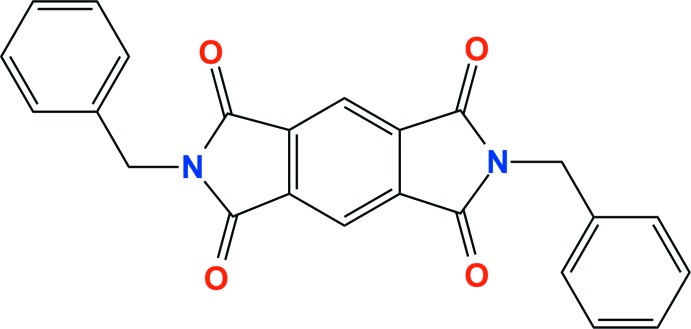


## Supra­molecular features   

**Figure 2:**
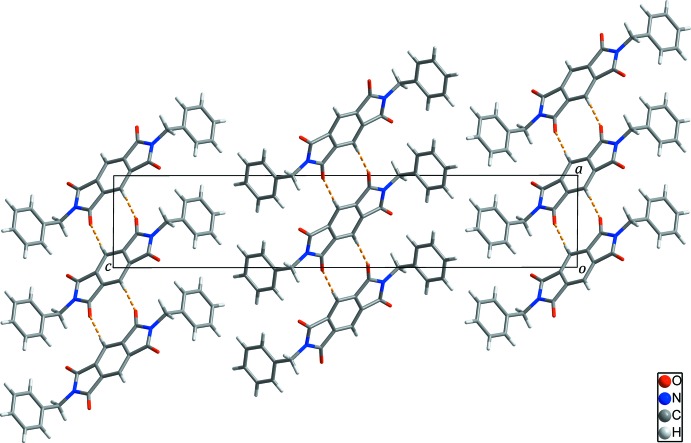
Chains of the title compound formed through inter­molecular C—H⋯O hydrogen bonds (yellow dashed lines).

**Figure 3:**
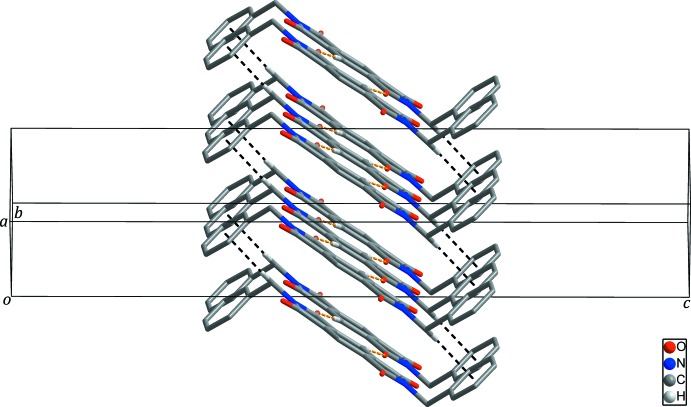
Supra­molecular layers of the title compound formed through inter­molecular C—H⋯π inter­actions (black dashed lines) between the chains generated by inter­molecular C—H⋯O hydrogen bonds (yellow dashed lines). H atoms not involved in inter­molecular inter­actions have been omitted for clarity.

In the crystal, adjacent mol­ecules are connected by weak C12—H12⋯O2 hydrogen bonds, Table 1 ▸ (yellow dashed lines in Fig. 2 ▸), forming inversion dimers. Inversion symmetry links these into a chain propagating along [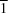
10]. Neighboring chains are linked through inter­molecular C—H⋯π inter­actions between a methyl­ene H atom and the terminal phenyl ring, resulting in the formation of supra­molecular layers extending parallel to the *ab* plane (black dashed lines in Fig. 3 ▸ and Table 1 ▸). These layers are separated from each other by 3.104 (3) Å. No inter­molecular π–π inter­actions are found between the pyromellitic di­imide moieties.

## Synthesis and crystallization   

The title compound was synthesized by the reaction of pyromellitic dianhydride with 2-phenyl­ethyl­amine according to a literature procedure (Kang *et al.*, 2015 ▸). X-ray quality single crystals were obtained by slow evaporation of a di­chloro­methane solution of the title compound.

## Refinement   

Crystal data, data collection and structure refinement details are summarized in Table 2 ▸. All H atoms were positioned geometrically with *d*(C—H) = 0.95 Å for C*sp* ^2^—H and 0.99 Å for methyl­ene, and were refined as riding with *U*_iso_(H) = 1.2*U*_eq_(C).

## Supplementary Material

Crystal structure: contains datablock(s) I, New_Global_Publ_Block. DOI: 10.1107/S2056989016017710/sj5513sup1.cif

Structure factors: contains datablock(s) I. DOI: 10.1107/S2056989016017710/sj5513Isup2.hkl

Supporting information file. DOI: 10.1107/S2056989016017710/sj5513Isup3.cml

CCDC reference: 1515263

Additional supporting information: crystallographic information; 3D view; checkCIF report

## Figures and Tables

**Table 1:** Hydrogen-bond geometry (Å, °) *Cg*1 is the centroid of the C1–C6 ring.

*D*—H⋯*A*	*D*—H	H⋯*A*	*D*⋯*A*	*D*—H⋯*A*
C7—H7*B*⋯O1	0.99	2.53	2.917 (2)	103
C12—H12⋯O2^i^	0.95	2.45	3.401 (2)	178
C7—H7*B*⋯*Cg*1^ii^	0.99	2.60	3.478 (2)	148

Symmetry codes: (i) 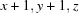; (ii) 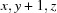.

**Table 2:** Experimental details

Crystal data	
Chemical formula	C_24_H_16_N_2_O_4_
*M*_r_	396.39
Crystal system, space group	Monoclinic, *P*2_1_/*n*
Temperature (K)	173
*a*, *b*, *c* (Å)	6.1500 (5), 4.7475 (3), 31.002 (2)
β (°)	90.461 (3)
*V* (Å^3^)	905.14 (11)
*Z*	2
Radiation type	Mo *K*α
μ (mm^−1^)	0.10
Crystal size (mm)	0.50 × 0.06 × 0.02

Data collection	
Diffractometer	Bruker APEXII CCD
Absorption correction	Multi-scan (*SADABS*; Bruker 2013 ▸)
*T*_min_, *T*_max_	0.661, 0.746
No. of measured, independent and observed [*I* > 2σ(*I*)] reflections	4593, 2016, 1444
*R*_int_	0.034
(sin θ/λ)_max_ (Å^−1^)	0.650

Refinement	
*R*[*F* ^2^ > 2σ(*F* ^2^)], *wR*(*F* ^2^), *S*	0.048, 0.119, 1.04
No. of reflections	2016
No. of parameters	136
H-atom treatment	H-atom parameters constrained
Δρ_max_, Δρ_min_ (e Å^−3^)	0.25, −0.22

Computer programs: *APEX2* and *SAINT* (Bruker, 2013 ▸), *SHELXS97* and *SHELXTL* (Sheldrick 2008 ▸), *SHELXL2014* (Sheldrick, 2015 ▸) and *DIAMOND* (Brandenburg, 2010 ▸).

## supplementary crystallographic information

### Crystal data

**Table:**

C_24_H_16_N_2_O_4_	*F*(000) = 412
*M**_r_* = 396.39	*D*_x_ = 1.454 Mg m^−^^3^
Monoclinic, *P*2_1_/*n*	Mo *K*α radiation, λ = 0.71073 Å
*a* = 6.1500 (5) Å	Cell parameters from 874 reflections
*b* = 4.7475 (3) Å	θ = 2.6–24.8°
*c* = 31.002 (2) Å	µ = 0.10 mm^−^^1^
β = 90.461 (3)°	*T* = 173 K
*V* = 905.14 (11) Å^3^	Needle, colourless
*Z* = 2	0.50 × 0.06 × 0.02 mm

### Data collection

**Table:**

Bruker APEXII CCD diffractometer	1444 reflections with *I* > 2σ(*I*)
φ and ω scans	*R*_int_ = 0.034
Absorption correction: multi-scan (SADABS; Bruker 2013)	θ_max_ = 27.5°, θ_min_ = 1.3°
*T*_min_ = 0.661, *T*_max_ = 0.746	*h* = −6→7
4593 measured reflections	*k* = −2→6
2016 independent reflections	*l* = −38→40

### Refinement

**Table:**

Refinement on *F*^2^	0 restraints
Least-squares matrix: full	Hydrogen site location: inferred from neighbouring sites
*R*[*F*^2^ > 2σ(*F*^2^)] = 0.048	H-atom parameters constrained
*wR*(*F*^2^) = 0.119	*w* = 1/[σ^2^(*F*_o_^2^) + (0.0529*P*)^2^ + 0.089*P*] where *P* = (*F*_o_^2^ + 2*F*_c_^2^)/3
*S* = 1.04	(Δ/σ)_max_ < 0.001
2016 reflections	Δρ_max_ = 0.25 e Å^−^^3^
136 parameters	Δρ_min_ = −0.22 e Å^−^^3^

### Special details

**Table:**

Geometry. All esds (except the esd in the dihedral angle between two l.s. planes) are estimated using the full covariance matrix. The cell esds are taken into account individually in the estimation of esds in distances, angles and torsion angles; correlations between esds in cell parameters are only used when they are defined by crystal symmetry. An approximate (isotropic) treatment of cell esds is used for estimating esds involving l.s. planes.

### Fractional atomic coordinates and isotropic or equivalent isotropic displacement parameters (Å^2^)

**Table:**

	*x*	*y*	*z*	*U*_iso_*/*U*_eq_	
O1	0.9145 (2)	0.9617 (3)	0.09759 (4)	0.0314 (4)	
O2	0.4737 (2)	0.2514 (3)	0.04921 (4)	0.0282 (3)	
N1	0.6589 (2)	0.6176 (3)	0.08217 (5)	0.0218 (4)	
C1	0.2783 (3)	0.3279 (4)	0.15445 (6)	0.0303 (5)	
H1	0.1617	0.3659	0.1350	0.036*	
C2	0.2505 (4)	0.1311 (4)	0.18711 (7)	0.0363 (5)	
H2	0.1157	0.0354	0.1901	0.044*	
C3	0.4206 (4)	0.0760 (4)	0.21524 (7)	0.0399 (6)	
H3	0.4021	−0.0568	0.2378	0.048*	
C4	0.6175 (4)	0.2130 (4)	0.21071 (6)	0.0356 (5)	
H4	0.7346	0.1724	0.2299	0.043*	
C5	0.6439 (3)	0.4094 (4)	0.17812 (6)	0.0298 (5)	
H5	0.7792	0.5036	0.1751	0.036*	
C6	0.4739 (3)	0.4699 (4)	0.14973 (6)	0.0233 (4)	
C7	0.4994 (3)	0.6895 (4)	0.11523 (6)	0.0252 (4)	
H7A	0.3565	0.7201	0.1011	0.030*	
H7B	0.5432	0.8691	0.1290	0.030*	
C8	0.8499 (3)	0.7687 (3)	0.07548 (6)	0.0213 (4)	
C9	0.9507 (3)	0.6455 (3)	0.03618 (5)	0.0199 (4)	
C10	0.8168 (3)	0.4283 (3)	0.02169 (5)	0.0186 (4)	
C11	0.6279 (3)	0.4093 (3)	0.05118 (5)	0.0211 (4)	
C12	1.1389 (3)	0.7249 (3)	0.01520 (5)	0.0202 (4)	
H12	1.2303	0.8725	0.0253	0.024*	

### Atomic displacement parameters (Å^2^)

**Table:**

	*U*^11^	*U*^22^	*U*^33^	*U*^12^	*U*^13^	*U*^23^
O1	0.0344 (9)	0.0305 (7)	0.0293 (7)	−0.0060 (6)	0.0023 (6)	−0.0098 (6)
O2	0.0247 (8)	0.0285 (7)	0.0316 (7)	−0.0056 (6)	0.0030 (6)	−0.0014 (6)
N1	0.0230 (9)	0.0219 (7)	0.0204 (8)	0.0001 (6)	0.0034 (6)	−0.0008 (6)
C1	0.0264 (11)	0.0300 (9)	0.0346 (11)	0.0002 (8)	0.0036 (9)	−0.0041 (9)
C2	0.0402 (14)	0.0287 (10)	0.0401 (12)	−0.0023 (10)	0.0169 (10)	−0.0015 (10)
C3	0.0580 (17)	0.0307 (10)	0.0313 (12)	0.0042 (11)	0.0151 (11)	0.0035 (9)
C4	0.0460 (14)	0.0341 (10)	0.0267 (10)	0.0039 (10)	−0.0045 (9)	0.0039 (9)
C5	0.0301 (12)	0.0315 (9)	0.0279 (10)	−0.0028 (9)	−0.0009 (9)	−0.0003 (9)
C6	0.0258 (11)	0.0228 (8)	0.0214 (9)	0.0009 (8)	0.0049 (8)	−0.0046 (7)
C7	0.0248 (11)	0.0269 (9)	0.0240 (9)	0.0025 (8)	0.0043 (8)	−0.0009 (8)
C8	0.0227 (10)	0.0198 (8)	0.0214 (9)	0.0011 (7)	−0.0010 (8)	0.0016 (7)
C9	0.0211 (10)	0.0184 (8)	0.0202 (9)	0.0011 (7)	−0.0025 (7)	0.0010 (7)
C10	0.0187 (9)	0.0181 (7)	0.0190 (8)	0.0005 (7)	0.0001 (7)	0.0024 (7)
C11	0.0239 (10)	0.0194 (8)	0.0199 (9)	0.0012 (8)	−0.0021 (7)	0.0027 (7)
C12	0.0229 (11)	0.0182 (7)	0.0195 (9)	−0.0014 (7)	−0.0019 (7)	0.0005 (7)

### Geometric parameters (Å, º)

**Table:**

O1—C8	1.210 (2)	C4—H4	0.9500
O2—C11	1.210 (2)	C5—C6	1.391 (2)
N1—C11	1.391 (2)	C5—H5	0.9500
N1—C8	1.393 (2)	C6—C7	1.503 (2)
N1—C7	1.465 (2)	C7—H7A	0.9900
C1—C6	1.387 (3)	C7—H7B	0.9900
C1—C2	1.389 (3)	C8—C9	1.491 (2)
C1—H1	0.9500	C9—C12	1.385 (2)
C2—C3	1.382 (3)	C9—C10	1.392 (2)
C2—H2	0.9500	C10—C12^i^	1.384 (2)
C3—C4	1.383 (3)	C10—C11	1.487 (3)
C3—H3	0.9500	C12—C10^i^	1.384 (2)
C4—C5	1.385 (3)	C12—H12	0.9500
			
C11—N1—C8	111.98 (15)	N1—C7—C6	114.25 (14)
C11—N1—C7	124.05 (15)	N1—C7—H7A	108.7
C8—N1—C7	123.68 (14)	C6—C7—H7A	108.7
C6—C1—C2	121.05 (19)	N1—C7—H7B	108.7
C6—C1—H1	119.5	C6—C7—H7B	108.7
C2—C1—H1	119.5	H7A—C7—H7B	107.6
C3—C2—C1	119.4 (2)	O1—C8—N1	125.37 (17)
C3—C2—H2	120.3	O1—C8—C9	128.58 (17)
C1—C2—H2	120.3	N1—C8—C9	106.04 (14)
C2—C3—C4	120.40 (19)	C12—C9—C10	122.98 (16)
C2—C3—H3	119.8	C12—C9—C8	129.19 (15)
C4—C3—H3	119.8	C10—C9—C8	107.81 (16)
C3—C4—C5	119.9 (2)	C12^i^—C10—C9	122.44 (16)
C3—C4—H4	120.1	C12^i^—C10—C11	129.49 (16)
C5—C4—H4	120.1	C9—C10—C11	108.03 (15)
C4—C5—C6	120.59 (19)	O2—C11—N1	125.35 (18)
C4—C5—H5	119.7	O2—C11—C10	128.50 (16)
C6—C5—H5	119.7	N1—C11—C10	106.14 (15)
C1—C6—C5	118.72 (17)	C10^i^—C12—C9	114.59 (15)
C1—C6—C7	120.50 (17)	C10^i^—C12—H12	122.7
C5—C6—C7	120.77 (17)	C9—C12—H12	122.7
			
C6—C1—C2—C3	−0.1 (3)	N1—C8—C9—C12	178.00 (17)
C1—C2—C3—C4	−0.7 (3)	O1—C8—C9—C10	−179.63 (17)
C2—C3—C4—C5	0.8 (3)	N1—C8—C9—C10	−0.36 (18)
C3—C4—C5—C6	−0.2 (3)	C12—C9—C10—C12^i^	−0.4 (3)
C2—C1—C6—C5	0.7 (3)	C8—C9—C10—C12^i^	178.05 (15)
C2—C1—C6—C7	−177.86 (17)	C12—C9—C10—C11	−178.18 (15)
C4—C5—C6—C1	−0.6 (3)	C8—C9—C10—C11	0.31 (18)
C4—C5—C6—C7	178.03 (17)	C8—N1—C11—O2	−178.89 (16)
C11—N1—C7—C6	71.0 (2)	C7—N1—C11—O2	−4.8 (3)
C8—N1—C7—C6	−115.62 (18)	C8—N1—C11—C10	−0.08 (18)
C1—C6—C7—N1	−115.50 (19)	C7—N1—C11—C10	173.99 (14)
C5—C6—C7—N1	65.9 (2)	C12^i^—C10—C11—O2	1.1 (3)
C11—N1—C8—O1	179.57 (16)	C9—C10—C11—O2	178.61 (17)
C7—N1—C8—O1	5.5 (3)	C12^i^—C10—C11—N1	−177.67 (16)
C11—N1—C8—C9	0.27 (18)	C9—C10—C11—N1	−0.15 (18)
C7—N1—C8—C9	−173.83 (14)	C10—C9—C12—C10^i^	0.4 (3)
O1—C8—C9—C12	−1.3 (3)	C8—C9—C12—C10^i^	−177.73 (16)

Symmetry code: (i) −*x*+2, −*y*+1, −*z*.

### Hydrogen-bond geometry (Å, º)

*Cg*1 is the centroid of the C1–C6 ring.

**Table:**

*D*—H···*A*	*D*—H	H···*A*	*D*···*A*	*D*—H···*A*
C7—H7*B*···O1	0.99	2.53	2.917 (2)	103
C12—H12···O2^ii^	0.95	2.45	3.401 (2)	178
C7—H7*B*···*Cg*1^iii^	0.99	2.60	3.478 (2)	148

Symmetry codes: (ii) *x*+1, *y*+1, *z*; (iii) *x*, *y*+1, *z*.
